# Evaluation of a Digital Handheld Hydrogen Breath Monitor to Diagnose Lactose Malabsorption: Interventional Crossover Study

**DOI:** 10.2196/33009

**Published:** 2021-10-18

**Authors:** Simon C Mathews, Sandy Templeton, Stephanie K Taylor, Sten Harris, Margaret Stewart, Shruti M Raja

**Affiliations:** 1 Johns Hopkins Medicine Baltimore, MD United States; 2 Electronics Program Penn Foster College Scottsdale, AZ United States; 3 Duke Early Phase Clinical Research Unit Durham, NC United States

**Keywords:** digital health, lactose intolerance, digestive disease, evaluation, medical device, detection, diagnostic, digestion, testing, performance, gastrointestinal, diagnosis

## Abstract

**Background:**

Lactose malabsorption is a common condition that affects a broad segment of the population. Clinical diagnosis based on symptom recall can be unreliable and conventional testing can be inconvenient, requiring expensive laboratory-based equipment and conduction of the testing in a clinical setting.

**Objective:**

The aim of this study is to assess the performance of a digital handheld hydrogen breath monitor (GIMate) in diagnosing lactose malabsorption compared to a US Food and Drug Administration (FDA)–cleared device (H2 Check) for the same indication.

**Methods:**

An interventional crossover study was performed in adult participants with a prior confirmed diagnosis of lactose malabsorption or a suspected history of lactose intolerance.

**Results:**

A total of 31 participants (mean age 33.9 years) were enrolled in the study. There was 100% positive percent agreement and 100% negative percent agreement between the GIMate monitor and the H2 Check. Correlation between gastrointestinal symptoms and hydrogen values was positive at 0.82 (*P*<.001).

**Conclusions:**

The digital handheld GIMate breath monitor achieved equivalent diagnostic performance to that of an FDA-cleared device in the diagnosis of lactose malabsorption.

**Trial Registration:**

ClinicalTrials.gov NCT04754724; https://clinicaltrials.gov/ct2/show/NCT04754724

## Introduction

Lactose malabsorption is a common condition due to lactase deficiency; for many, it results in gastrointestinal symptoms, which is termed lactose intolerance [1]. Lactase is an enzyme occurring in the intestinal mucosa that hydrolyzes lactose into its constituent parts, galactose and glucose [2]. The enzyme is normally present in neonates; however, for a majority of individuals worldwide, there is an inherited and irreversible reduction in enzyme activity as individuals age [3]. Secondary lactose malabsorption can also occur when there is injury to the intestinal mucosa from a reversible condition, such as infection [4]. Not all individuals with lactose malabsorption will experience bothersome gastrointestinal symptoms (ie, lactose intolerance). Those with lactose intolerance are often diagnosed clinically—that is, their response is observed to lactose challenges that come through dietary exposure, followed by a trial of avoidance of lactose-containing products [5]. However, self-reported intolerance can often be unreliable [6,7], and formal diagnosis is still helpful in many cases.

Conventional testing is conducted through a hydrogen breath test in the ambulatory clinical setting. Individuals usually present to the testing site fasting, and a baseline breath reading is obtained. They are subsequently challenged with a lactose-containing solution, with follow-up hydrogen readings obtained on an hourly basis for 3 hours [8].

The mechanism of hydrogen detection is based on undigested lactose in the colon being fermented by bacteria and resulting in the production of hydrogen, which is then partially absorbed into the bloodstream and ultimately exhaled by the lungs via the pulmonary circulation and gas exchange. Direct lactase activity can also be measured on tissue obtained through jejunal biopsy via endoscopy. This approach, however, is more invasive, costly, and potentially less reliable given issues relating to sampling bias [5].

Current methods for hydrogen breath testing can be costly for physicians to purchase and inconvenient for patients because they must take time out of their day to go to the testing site for several hours. In addition, given that most conventional breath-testing equipment is reusable, infectious contamination can occur through aerosolized breath contents. As a result, the validation of an alternative breath test that is portable, handheld, and disposable presents an opportunity to improve the value, safety, and experience associated with hydrogen breath testing.

In this study, our primary aim was to compare the performance of the GIMate (Vivante Health), a novel digital handheld hydrogen breath monitor, to that of the H2 Check (Micro Medical Limited), a device cleared by the US Food and Drug Administration (FDA) for the diagnosis of lactose malabsorption.

## Methods

### Study Design and Procedure

The study was an interventional crossover design, with all participants receiving both diagnostic interventions. The order of which intervention was received first was alternately assigned at random (Multimedia Appendix 1). The study was conducted at Duke University and was reviewed and approved by the Duke University Institutional Review Board. Upon screening as eligible for the study, participants were provided with best practice pre–breath testing guidance [9], including dietary restrictions and fasting overnight. Baseline breath hydrogen measurements were performed the following day using the GIMate and H2 Check. This was followed by ingestion of a 25 g lactose solution and subsequent measurement of breath hydrogen on both devices at 1-hour, 2-hour, and 3-hour time points. During each of these measurements, participants completed a Likert-scale assessment of gastrointestinal symptom severity (0, none; 1, mild; 2, moderate; 3, moderately severe; 4, severe; 5, very severe).

### Patient Population

Adults aged 18-55 years with a self-reported history of lactose malabsorption or lactose intolerance were recruited. Exclusion criteria included history of prior gastrointestinal surgery; self-reported history of any chronic gastrointestinal disease (eg, gastroesophageal reflux disease, celiac disease, Crohn disease, ulcerative colitis, pancreatitis); self-reported history of endocrine or metabolic disease that may impact gastrointestinal or colonic function (eg, hyper/hypothyroidism, diabetes); clinically significant cardiovascular, respiratory, renal, hepatic, hematologic, neurologic, or psychiatric disease for which chronic therapy (prescription or nonprescription) is required; self-reported history of allergic reaction to any drug or drug component; antibiotic use within 28 days of lactose malabsorption testing; use of nonantibiotic prescription or over-the-counter products (dietary or digestive supplements and laxatives) within 14 days of testing; self-reported use of nicotine-containing products or chronic secondhand smoke exposure within 14 days of testing; pregnancy; any other condition which in the Investigator’s opinion may adversely affect the participant’s ability to complete the study or its measures or which may pose significant risk to the participant based on medical history or physical examination; and consumption of food after midnight on the day of testing (within 12 hours) of testing or consumption of a nonwater beverage after midnight (or less than 8 hours) prior to testing.

### Devices

**Figure 1:**
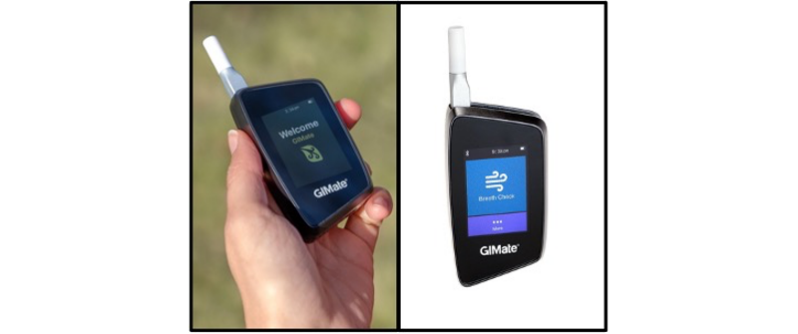
The GIMate digital hydrogen monitor.

The Micro H2 is a hydrogen monitor that has been cleared by the FDA [10] for the diagnosis of lactose malabsorption using an automatic sensor drift detection approach, which requires gas calibration [7]. The GIMate is a portable, handheld digital hydrogen monitor with a touch screen interface that detects hydrogen using a metal-oxide sensor, which does not require calibration (Figure 1). Results from breath tests are displayed to users on the digital touch screen and stored on the device.

### Outcome Measures

The primary outcome measure was positive percent agreement (PPA) in the diagnosis of lactose malabsorption of the GIMate compared to the H2 Check. Additional outcome measures included negative percent agreement (NPA) and correlation between GIMate hydrogen levels and patient self-reported gastrointestinal symptom severity. The protocol for measuring hydrogen levels was consistent with recent guidelines with a baseline measurement performed, followed by consumption of a 25 g lactose solution with subsequent hourly breath hydrogen measurements for a 3-hour period using both monitors. A positive diagnosis of lactose malabsorption was defined as a breath hydrogen level increase by 20 ppm or more from baseline at any point during the 3-hour measurement period based on guidelines [8]. A secondary outcome was the correlation between self-reported gastrointestinal symptoms and GIMate hydrogen readings.

### Statistical Calculations and Analyses

The calculation for PPA was (number of individuals diagnosed with lactose malabsorption with GIMate) / (total number of individuals diagnosed with lactose malabsorption with H2 Check). The calculation for NPA was (number of individuals negative for lactose malabsorption with GIMate) / (total number of individuals negative for lactose malabsorption with H2 Check). Correlations between gastrointestinal symptoms and GIMate readings were calculated using the two-sided Spearman rank-based correlation measure of association. The *P* value was computed for testing for correlation estimate=0.

## Results

### Population Demographics

A total of 39 individuals were screened; of these, 31 were eligible to complete the lactose challenge. Demographic characteristics for the study participants are included in Table 1. The 8 participants who were excluded from the study did not meet the eligibility criteria, including no self-reported history of lactose malabsorption or intolerance (n=1); history of prior gastrointestinal surgery (n=1); history of chronic gastrointestinal disease (n=1); clinically significant condition requiring ongoing therapy (n=1); history of allergic drug reaction (n=2); and investigator determination of a condition that would pose unnecessary risk or would adversely affect participation in the study (n=2: 1 participant had a milk allergy, and 1 participant was unable to participate during normal work hours).

**Table 1:** Demographics of the study participants (N=31).

Characteristic		Value
Age (years), mean (SD)		33.9 (7.3)
**Sex, n (%)**		
	Female	17 (55)
	Male	14 (45)
**Race, n (%)**		
	Asian	3 (10)
	Black/African-American	14 (45)
	White/Caucasian	14 (45)
**Ethnicity, n (%)**		
	Hispanic or Latino	3 (10)
	Non-Hispanic or non-Latino	28 (90)

### Outcomes

The results for the primary outcomes regarding diagnostic performance are detailed in Table 2, demonstrating a PPA of 100% and an NPA of 100%.

**Figure 2:**
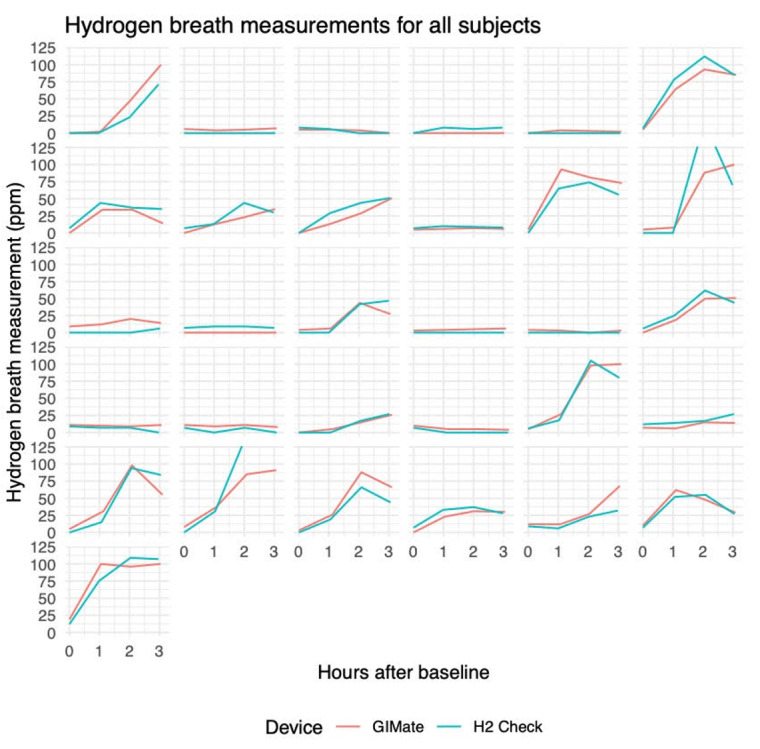
GIMate and H2 Check hydrogen breath measurements by participant.

The relationship between GIMate and H2 Check on a per-subject basis is depicted in Figure 2.

The relationships between GIMate and H2 Check readings for all participants across all time periods are demonstrated in Multimedia Appendix 2.

The secondary outcome of correlation between gastrointestinal symptoms and GIMate hydrogen is detailed in Multimedia Appendix 3, demonstrating an overall Spearman rank correlation of 0.82 across all time periods.

**Table 2:** Lactose malabsorption diagnosis by detection method (N=31).

	H2 Check test results	
GIMate test results	Positive	Negative
Positive	18	0
Negative	0	13
Total	18	13

## Discussion

### Principal Results

The primary finding of this study is the 100% positive and negative percent agreement between GIMate and H2 Check for the diagnosis of lactose malabsorption, indicating equivalent diagnostic performance between both devices. This finding was also supported by the close relationship between individual hydrogen readings on both devices for each participant (Figure 1). The correlation estimate of 0.82 between GIMate hydrogen readings and gastrointestinal symptoms indicated a strong relationship between these two variables. This finding also suggests that the rise in hydrogen levels was likely associated with lactose intolerance in our study population, which is diagnosed based on gastrointestinal symptoms in the presence of lactose malabsorption. The highest correlation between symptoms and hydrogen levels was seen at the 2-hour interval, which suggests that symptom response was greatest prior to the end of formal testing at the 3-hour mark.

Prior studies examining portable hydrogen breath testing have been conducted, including a study focused on 29 adult and pediatric patients, which required a nasal prong and syringe to obtain samples and specifically included participants with comorbid gastrointestinal conditions (eg, irritable bowel syndrome, bacterial overgrowth) [11]. Another study included 12 patients with suspected lactose intolerance using a proprietary score calculated in “arbitrary units” and did not include diagnostic criteria for lactose malabsorption [12]. This study also required the use of a phone app to display data. The H2 Check was previously evaluated in 44 patients (77% female) and compared to a composite gold standard assessment that included breath, blood, and urine testing [7]. Prior studies did not conform to the 2017 consensus guidelines on hydrogen breath testing, which provided best practice recommendations on key experimental elements such as dose of lactose, frequency of breath testing interval, and diagnostic criteria.

As a result, this study was the first to validate a portable, handheld device in diagnosing lactose malabsorption using the strongest and most current evidence-based approach. In addition, it demonstrated the first completely digital and portable measurement of breath hydrogen using clinically validated endpoints in a standalone, handheld device. This study also used the most stringent eligibility criteria compared to prior studies to minimize the risk of confounding due to the impact of comorbid gastrointestinal, surgical, and medical conditions on the production and detection of hydrogen. In addition, it is the first study to compare a novel breath hydrogen device to a previously FDA-cleared device in diagnostic performance for lactose malabsorption.

Lastly, while this was not explicitly evaluated, the compact, digital interface and substantially lower cost of the GIMate make it a potentially more convenient and safer alternative to conventional testing because it can be discarded after single-person use. These features also highlight its potential application in nonclinical settings, including home use. In this context, written instructions or integrated decision support could alert patients as to when to contact their physician based on the results. Although our study was the first to demonstrate the digital transformation of hydrogen breath testing, other studies have examined digital breath testing of other gases, including carbon dioxide [13], carbon monoxide [14], and hydrogen peroxide [15].

Limitations of this study included its relatively small sample size, although it was comparable to that in prior studies and included a more balanced and diverse representation of the population. In addition, our study was the first to examine a US-based population using a portable device. An additional limitation was the inability to directly compare our results with prior studies of portable breath hydrogen measurement, as prior studies used heterogeneous testing methods that did not conform to the most recent guidelines (two of the three studies were published prior to the establishment of these guidelines).

### Conclusions

This study demonstrated that the GIMate has equivalent diagnostic performance to the H2 Check in the diagnosis of lactose malabsorption. It represents the first entirely digital approach to diagnosing lactose malabsorption with a portable, handheld device using validated clinical endpoints. These findings indicate that the GIMate is a potential viable alternative for portable, handheld detection of lactose malabsorption.

## Appendix

Multimedia Appendix 1 CONSORT (Consolidated Standards of Reporting Trials) diagram.

Multimedia Appendix 2 Scatterplot of all GIMate and H2 Check readings.

Multimedia Appendix 3 Spearman rank correlations between gastrointestinal symptoms and GIMate readings.

## Abbreviations

FDA: US Food and Drug Administration
NPA: negative percent agreement
PPA: positive percent agreement
