# Supplementary figures and images for: Evaluation of a Digital Handheld Hydrogen Breath Monitor to Diagnose Lactose Malabsorption: Interventional Crossover Study
Source: JMIR Form Res. 2021 Oct 18;5(10):e33009. doi: 10.2196/33009 (PMC8561400; doi:10.2196/33009)

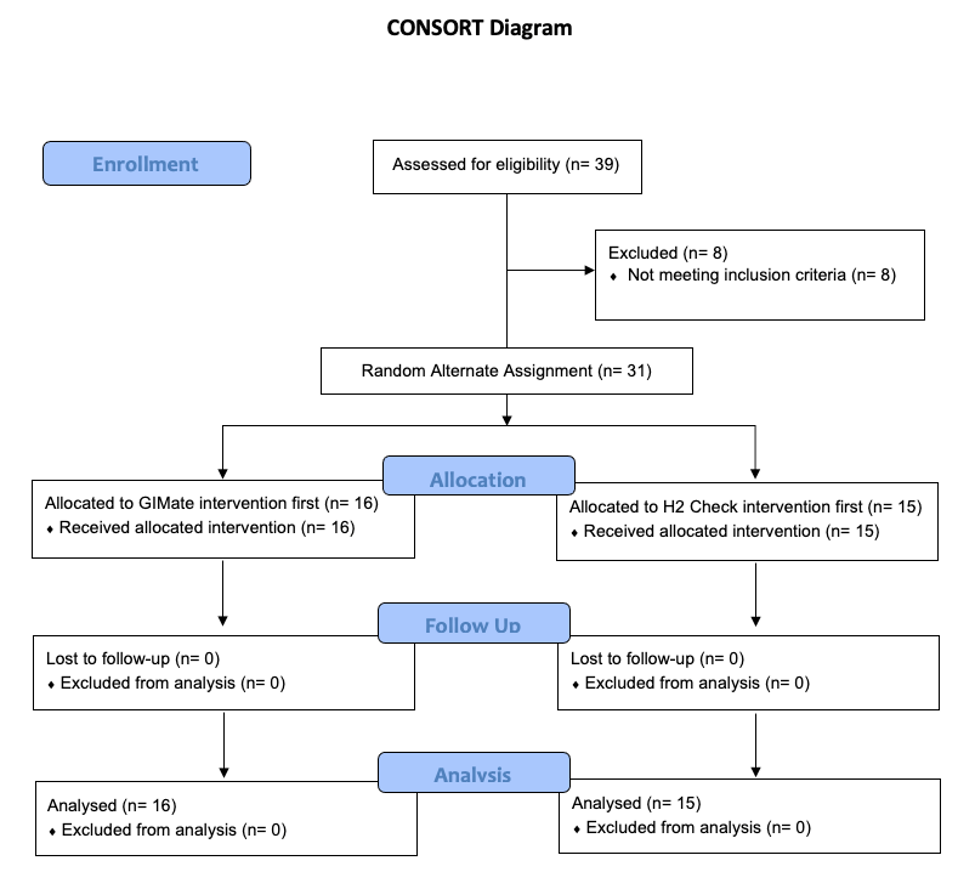

Supplement: Multimedia Appendix 1 [file formative_v5i10e33009_app1.png]

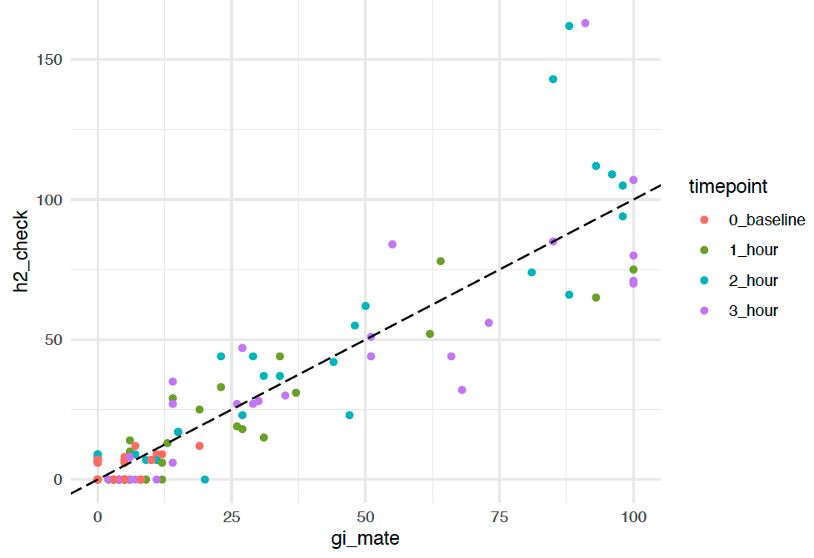

Supplement: Multimedia Appendix 2 [file formative_v5i10e33009_app2.png]

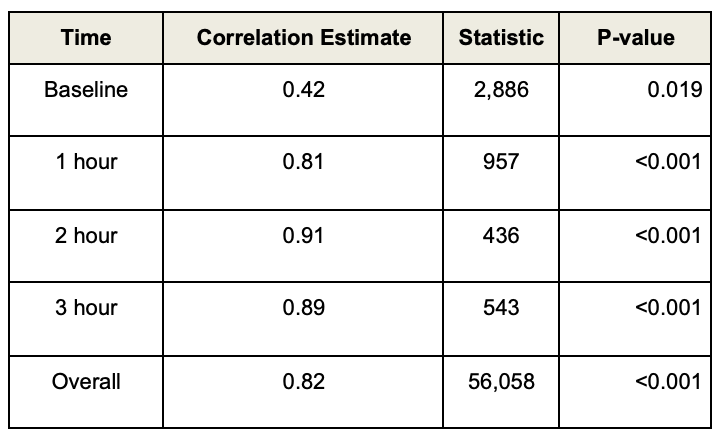

Supplement: Multimedia Appendix 3 [file formative_v5i10e33009_app3.png]
